# The Use of Extracellular Membrane Vesicles for Immunization against Francisellosis in Nile Tilapia (*Oreochromis niloticus*) and Atlantic Cod (*Gadus morhua* L.)

**DOI:** 10.3390/vaccines9010034

**Published:** 2021-01-09

**Authors:** Verena Mertes, Alexander Kashulin Bekkelund, Leidy Lagos, Elia Ciani, Duncan Colquhoun, Hanne Haslene-Hox, Håvard Sletta, Henning Sørum, Hanne Cecilie Winther-Larsen

**Affiliations:** 1 Section of Pharmacology and Pharmaceutical Biosciences and Centre of Integrative Microbial Evolution, Department of Pharmacy, University of Oslo, 0315 Oslo, Norway; verename@uio.no (V.M.); leidy.lagos@nmbu.no (L.L.); elia.ciani@farmasi.uio.no (E.C.); 2 Department of Food Safety and Infection Biology, Norwegian University of Life Sciences, 1430 Ås, Norway; a.bekkelund@creek-bio.com (A.K.B.); henning.sorum@nmbu.no (H.S.); 3 Fish Health Research Group, Norwegian Veterinary Institute, 4045 Oslo, Norway; duncan.colquhoun@vetinst.no; 4 Department of Biotechnology and Nanomedicine, SINTEF Industry, 7034 Trondheim, Norway; Hanne.Haslene-Hox@sintef.no (H.H.-H.); Havard.Sletta@sintef.no (H.S.)

**Keywords:** francisellosis, vaccine, membrane vesicles, fish disease, Atlantic cod, tilapia

## Abstract

Francisellosis in fish is caused by the facultative intracellular Gram-negative bacterial pathogens *Francisella noatunensis* ssp. *noatunensis* and *Francisella orientalis*. The disease is affecting both farmed and wild fish worldwide and no commercial vaccines are currently available. In this study, we tested isolated membrane vesicles (MVs) as possible vaccine candidates based on previous trials in zebrafish (*Danio rerio*) indicating promising vaccine efficacy. Here, the MV vaccine-candidates were tested in their natural hosts, Atlantic cod (*Gadus morhua* L.) and Nile tilapia (*Oreochromis niloticus*). Injection of MVs did not display any toxicity or other negative influence on the fish and gene expression analysis indicated an influence on the host immune response. However, unlike in other tested fish species, a protective immunity following vaccine application and immunization period could not be detected in the Atlantic cod or tilapia. Further in vivo studies are required to achieve a better understanding of the development of immunological memory in different fish species.

## 1. Introduction

Members of the *Francisellaceae* are Gram-negative, aerobic, facultative intracellular cocco-bacilli with sizes ranging from 300 to 700 nm in diameter [1,2,3,4]. The francisella species causes infection in a range of hosts. Most subspecies within *Francisella tularensis* cause the disease tularemia, which can infect a number of mammals, including humans [5]. Other *Francisella* species, *F. noatunensis* ssp. *noatunensis* (*Fnn*), *F. noatunensis ssp. chilensis* and *F. orientalis* (*Fo*) (previously *F. noatunensis ssp. orientalis*) are adapted to the aquatic environment. *Fnn* and *Fo* cause francisellosis, a systemic infection in both farmed and wild fish living in cold or warm water, respectively [6,7,8,9,10,11,12].

*Francisella* infections in fish are mainly characterized by macroscopically visible granuloma on gills and skin as well as formation of internal granulomas, usually prominent in spleen, heart, kidney and liver [2,8,13]. Early reports of francisellosis caused by *Fnn* were registered after several Norwegian Atlantic cod *(Gadus morhua* L.) farms experienced losses of up to 40% between July and August 2005. The infection was described as a severe granulomatous inflammatory disease [2,3]. Systematic granulomatous conditions had been previously reported in cultured Nile tilapia (*Oreochromis niloticus*) (further referred to as tilapia) in Taiwan, Hawaii and the continental United States [6,14,15,16]. It was, however, only confirmed in 2007 that the *Fo* species was the causative agent of these outbreaks [17]. Infections in tilapia with *Fo* display a high mortality rate, whereas infection in Atlantic cod with *Fnn* is characterized as a chronic infection [13]. 

To date, there is no efficient vaccine against francisellosis available on the market. Vaccine trials using whole-cell inactivated bacteria with or without different oil adjuvants displayed only limited protection [18,19,20]. Attenuated live vaccines have demonstrated protection, but may be subject to licensing difficulties due to possible reversion back to virulence [21,22,23,24].

An alternative vaccine approach is the use of extracellular membrane vesicles (MVs) secreted by the bacteria [25,26,27]. The spherical MVs are 10–300 nm in diameter and contain different proteins, DNA, RNA and virulence factors and are thereby representing the mother cell in a nonreplicative form [27,28]. Vaccination with MVs has previously been shown to provide protection in a range of species [29,30]. In fish, MV immunization has provided protection against, i.e., edwardsiellosis infection in olive flounder (*Paralichthys olivaceus*) [31]. Moreover, immunization experiments against francisellosis using isolated MVs showed promising results towards protective immunity against *Fnn* [26] and *Fo* [27] in an adult zebrafish (*Danio rerio*) model. The immunization with *Fo* MVs induced an antibody response in zebrafish and infection challenge of vaccinated zebrafish displayed a 65.5% survival rate [27]. Considering the nonlethal chronic infection characteristics of *Fnn*, the vaccine derived from *Fnn* MVs, induced protection against subsequent *Fnn* infection in zebrafish [26]. Due to the protective effect of the MV immunization observed in zebrafish against infection from *Fnn* and *Fo*, in the current study the immunization properties of isolated MVs were tested in their natural hosts, the Atlantic cod and tilapia, respectively. 

## 2. Materials and Methods

### 2.1. Bacteria, Media and Growth Conditions

*Fnn* strain NCIMB14265^T^ was isolated from diseased Atlantic cod in Norway [2]. *Fo* strain 07-285A was an isolate from diseased tilapia in Costa Rica [32]. Cultivation was performed on ECA plates consisting of 30.4 g/L BD Bacto™ Eugon Broth (Difco Laboratories, US) supplemented with 15 g/L Microbiology Agar (Merck) and 5% bovine blood (Håtunlab AB) or in Eugon Broth (BD Bacto^TM^ Eugon Broth, Difco Laboratories, US) supplemented with 2 mM FeCl_3_ (Sigma-Aldrich, Saint Louis, MO, USA) with agitation (100 rpm) at 20 °C as previously described in [33,34]. Bacterial stocks were frozen in autoclaved 10% skimmed milk (Difco, Sparks, US) or in Bacto Eugon broth supplemented with 20% glycerol (Sigma-Aldrich, Saint Louis, MO, USA) and stored at −80 °C.

### 2.2. Isolation of Membrane Vesicles

*Fnn* and *Fo* MVs were produced in batch fermentation (3-L Applikon fermenters) at 20 °C in BD Bacto™ Eugon Broth (Difco Laboratories, Franklin Lakes, NJ, USA) supplemented with 2 mM FeCl_3_ (Sigma-Aldrich, Saint Louis, MO, USA), and pH was retained at 7.0. Dissolved oxygen was maintained at 20% with a flow of 15–24 VVh and stirring. The culture was inoculated at OD_600_ of 0.1 and harvested at OD_600_ of 4.0. The culture was centrifuged and the cell free supernatant was stored at −80 °C awaiting further purification of MVs. The frozen ferment was thawed and sterile filtered (0.2 µm, Nalgene Rapid-Flow Sterile Disposable Filter Units with PES Membrane, Cat. No: 124-0045, Thermo Fisher, Waltham, MA, USA) prior to further processing. Tangential flow filtration was used under sterile conditions to concentrate the MVs. The pump rate for filtration was 30–50 ml retentate/min at a premembrane pressure of 1.0–1.8 bars. Three membranes (Pellicon XL 50 cm^2^ Microfiltration Cassettes, Merck Millipore, Burlington, MA, USA) with cut-offs of 10 kDa were connected in parallel. The retentate (including MVs) was recirculated for ~3 h by recirculating the retentate flow to the feed solution. The concentrated fermentate was stored at 4 °C until ultracentrifugation (Sorvall 80 MX, (Thermo Scientific, Waltham, MA, USA) with a fixed angle rotor T-865, average 110,000× *g*, 2 h, 20 °C). The supernatant was decanted immediately after centrifugation, and the pellet was washed two times with hydroxyethyl piperazineethanesulfonic acid (HEPES) buffer (50 mM, pH 6.8, Sigma-Aldrich, Saint Louis, MO, USA) and centrifuged (110,000× *g*, 1 h, 20 °C). The pelleted MVs were suspended in Phosphate Buffered Saline (PBS) solution. A small volume of purified MVs was spread on ECA plates (see Section 2.1) and Luria-Bertani (LB) agar plates (LB broth with agar, Sigma-Aldrich, Saint Louis, MO, USA) to ensure sterility. The protein concentration was measured with a Qubit 2.0 Fluorometer (Invitrogen by Life Technologies, San Diego, CA, USA) and used as an indirect estimate of MV concentration. MVs were frozen at −80 °C before use in vaccination trials.

### 2.3. Toxicity, Immunization and Challenge Trial in Tilapia

The toxicity of different concentrations of MVs was assessed in a pilot toxicity trial in tilapia. All experiments in tilapia were performed at the Norwegian University of Life Sciences, following the regulations controlling experiments with live animals in Norway and approved by the Norwegian Food Safety Authority, Oslo, Norway (FOTS ID 6966/7026). The experimental fish were held in recirculating aquaculture systems (RAS) in 250 L tanks (32 fish), at a water temperature of 25 °C, pH 7.4 with dissolved oxygen at least 5.4 mg/L or better; the experimental animals were fed daily (Aller Aqua, Christiansfeld, Denmark) at a rate of 2% bodyweight according to standard protocols as described in Soto et al. 2013 [10]. The fish were incubated with 12 h light and 12 h dark photoperiods and were monitored daily. Experiments were conducted on randomly chosen animals of both gender at an average weight of 25 g (fish were bulked weight); animals were marked with Radio Frequency Identification (RFID) tags (also called Passive integrated transponder (PIT) tags) [35].

The trial included four test groups, each consisting of eight fish. The different test groups were intraperitoneal (IP) injected with 100 µL physiological saline solution containing either 40 or 400 µg of MVs from *Fo* or physiological saline alone. One test group served as control where the fish remained untreated. Fourteen days after injection all fish were euthanized with 0.03 mg/L Benzoak^®^ 200 mg/mL (ACD Pharmaceuticals AS, Leknes, Norway). Gene expression levels were evaluated using harvested spleen tissue for quantitative PCR (qPCR) analysis. Additionally, serum samples were taken to evaluate *Fo* specific immunoglobulin (IgM) antibody level, using enzyme-linked immunosorbent assay (ELISA).

For the MV immunization trial in tilapia, an IP injection of 40 µg of MV suspended in 100 µL PBS, isolated from *Fo*, was administered. The fish had an average start weight of 25 g and were held and fed under the same conditions as described above for the toxicity experiment. The four experimental groups with 36 tilapia in each group were kept in perforated metal baskets with metal nets (pore size 2 mm) in the same 250 L glass fiber tank. The four experimental groups consisted of a MV vaccinated group injected with 100 µL of 40 µg of *Fo* MVs emulsified in the adjuvant Montanide ISA 761 VG; L10415 (Seppic), an adjuvant group, a placebo group (injected with 100 µL physiological saline) and a control group (did not receive any treatment). Each of the experimental groups consisted of 36 fish. After vaccination, the fish were held for 24 days (600 degree/days) to allow development of specific immunity. Following the immunization period, fish were subjected to cohabitation challenge with disease carriers (shedders; 29 fish) that were inoculated intraperitoneally with 100 µL of *Fo* (3 × 10^5^ CFU/mL) and kept in a separate tank of 250 L that was connected to the experimental tank with a recirculating pump introducing the *Fo* pathogen into the rearing water. Mortality was monitored for 61 days. For evaluation of immune response parameters, spleen and head kidney tissues were collected and used for qPCR analysis. Head kidney, heart and muscle tissues from survivors were collected after 61 days for later evaluation of bacterial burden by qPCR.

### 2.4. Toxicity, Immunization and Challenge in Atlantic Cod

All experiments in Atlantic cod were performed at Tromsø research station, Kårvika, following the regulations controlling experiments with live animals in Norway and approved by the Norwegian Food Safety Authority (FOTS ID 8171/8172). To exclude potential harmfulness of different concentrations of MVs isolated from *Fnn*, a toxicity trial was performed. The experimental groups were held together in one 300 L tank (32 fish). The fish were of mixed gender and were marked with RFID tags. During the duration of the experiments, the fish were maintained at 12 °C water temperature with fresh flow through marine water (1.5 L/kg/min) and dissolved oxygen was 8.0 mg/L or better. The fish were exposed to 12 h of artificial light and fed twice a day with dry pellets at a rate of 1% of the body weight (start weight 30 g). Two different doses of *Fnn* MVs (40 and 400 µg) were administered to two groups of fish by a single IP injection dissolved in 100 µL of physiological saline solution. Additionally, a placebo group (injected with 100 µL of physiological saline) and a control group (did not receive any treatment) were included in the toxicity trial. Each experimental group consisted of 8 fish. After 14 days, the fish were euthanized using a two-stage procedure suitable for fish with anesthesia using 0.03 mg/L Benzoak^®^ 200 mg/mL (ACD Pharmaceuticals AS, Leknes, Norway) followed by a sharp blow to the head. Spleen tissues from all groups were harvested and immune gene expression levels were evaluated using qPCR.

For the MV immunization trial in Atlantic cod, the fish were kept under the same conditions as described above and had a starting weight of 30 g (fish were bulk weight). The size of the tank the fish were kept in was 1800 L. All groups were held together in one tank (312 fish). Fish were randomly put into 4 groups of 65 individuals; each individual from the vaccine group received a single IP injection of 100 µL solution containing 40 µg MV conjugated with Montanide ISA 761 VG L10415 (Seppic) adjuvant. Individuals from the adjuvant group received an injection with Montanide ISA 761 VG L10415 only. Fish from the placebo group received a single IP injection of 100 µL of physiological saline. The fourth group was an untreated control group. Fifty days after injection, the fish were challenged using a cohabitation model using shedder fish (40 individuals), to allow natural disease transmittance between the groups. Shedders were inoculated by IP injection of the pathogen (3.0 × 10^7^ CFU/mL). The fish were observed for around 8 weeks (61 days) and random fish of each group were euthanized at the set time-point of 30 days postchallenge (dpc) (12 fish per group) and 61 dpc (24 fish per group) for sampling of head kidney, spleen and serum for experimental evaluation of immune gene expression level using qPCR.

### 2.5. Bacteriology

The liver and a block of tissue of randomly selected tilapia were aseptically collected and homogenized with steel beads in 500 µL of sterile 1% NaCl for 20 s to disrupt tissues. The suspension was briefly spun in a microcentrifuge and plated on Eugon Chocolate Agar (BD). Plates were incubated at 20 °C and observed for colonies. Colonies were verified with Gram staining (data not shown).

### 2.6. RNA Isolation and Quantitative Real-Time PCR

Tissues harvested for RNA isolation were stored in RNAlater (Ambion, Life Technologies, Carlsbad, CA, US) at −20 °C until further processing. Total RNA was extracted using the RNeasy kit (Qiagen, Hilden, Germany) according to the manufacturer’s instructions. Tissues were homogenized in 600 µL of RLT buffer (supplemented in RNeasy Plus Mini Kit, Qiagen) using glass beads (Sigma Aldrich) and homogenizer Precellys 24 (Bertin Technologies, Minilys, Montigny le Bretonneux, France). RNA was eluted in 30 µL RNAse-free H_2_O (Qiagen). RNA was quantified using a Nanodrop UV5Nano (Mettler Toledo, Greifensee, Switzerland). Reverse transcription was performed using a High Capacity RNA to cDNA kit (Applied Biosystems, Thermo Scientific, Vilnius, Lithuania) following the manufacturer’s protocol. Gene expression levels were analyzed via qPCR using a CFX89 lightcycler (Bio-Rad, Singapore). The reaction was carried out using either LightCycler® 480 SYBR Green I Master (Roche, Indianapolis, IN, US) or iTaq Universal SYBR Green Supermix (Bio-Rad, US), cDNA was diluted to 1:10 and the final reaction volume was 10 µL. Primers used are listed in Table A1 and qPCR was performed at 62 °C annealing temperature and 40 cycles. Relative expression (ΔΔC_t_) was calculated either using Bio-Rad CFX Maestro software or the spreadsheet from Vandesompele et al. [36]. Gene expression was normalized against one or two reference genes, respectively (*β-actin* in tilapia; *ef1α* and *ubiquitin* (Ubi) in Atlantic cod), whose stability was tested with the Bio-Rad CFX Maestro software. For Atlantic cod the following immune marker genes were measured: *il1β*, *il6*, *il8*, *il10*, *infγ*, *igm-lc*, *igm-hc*, while for tilapia *il1β*, *tnfα*, *cd83*, *igm*, *infγ*, *il12*, *cox2*, *mhcII*, *igd* immune marker genes were measured. All immune marker genes were tested for both the MV toxicity and the MV vaccination trial. Genes that did not display any detectable expression level were not included in the figures.

### 2.7. Serum Collection and ELISA

Serum samples from tilapia were collected from eight fish at the end of the toxicity trial. In the case of Atlantic cod, serum samples were collected from three fish from both the toxicity assay and from three fish from each group in the vaccine trial at the time-points 0, 30 and 61 dpc. Enzyme-linked immunosorbent assay (ELISA) was performed using an anti-Tilapia *(Oreochromis niloticus)* IgM monoclonal antibody (Aquatic Diagnostics Ltd., Stirling, Scotland) and anti-Cod (G. *morhua* L)/Haddock (*Melanogrammus aeglefinus*) IgM monoclonal antibody (Aquatic Diagnostics Ltd., Stirling, Scotland), following the manufacturer’s protocol.

### 2.8. Quantification of Bacterial Burden in Tilapia

The presence of bacterial genomic DNA (gDNA) in heart, head kidney and muscle tissues was evaluated using a primer pair specific for *Fo* [37] (Table A1). Sampled tissue was transferred to RNAlater (Ambion) and stored at 4 °C. Extraction of gDNA was performed with the DNeasy Blood and Tissue Kit (Qiagen) according to the manufacturer’s protocol; qPCR was performed with a volume of 20 µL and *Fo* gDNA was used as standard for the qPCR plate. 

### 2.9. Statistical Analysis

Statistical analyses were performed using GraphPad Prism software version 8. The data were checked for normal distribution (Shapiro–Wilk Normality test) and outliers were identified and removed using robust regression and outlier removal (ROUT) (Q = 1%). The assumption of equal variances (i.e., assumption of homoscedasticity) was tested using the Brown–Forsythe test and, if needed, the data were log-transformed to meet the test criteria. Statistically relevant differences in gene expression among treatments were assessed by one-way ANOVA, followed by a Tukey multiple comparison test. The survival curve was generated using the Kaplan–Meier method.

## 3. Results

### 3.1. Fo MV Toxicity and Immunization Trial in Tilapia

#### 3.1.1. High Concentration of Fo MVs Are Not Toxic and Elicit a Limited Immune Response in Tilapia

Before initiating the MV vaccine trial in tilapia, the toxicity of two different doses of MVs were tested. The 40 µg dose was chosen based on previous concentrations of MV used in immunization experiments in fish and mammals [27,31,38,39]. In addition, a ten times higher dose of 400 µg was used. Tilapia did not display any clinical signs or toxic reaction to either a low (40 µg) or high (400 µg) dose of injected MVs. During dissection, no macroscopically visible pathological effects were observed in the internal organs in response to the IP injection. To further evaluate differences in immune responses to different MV concentrations, gene expression levels were measured for a selection of immune-related genes (*il1β*, *tnfα*, *cd83*, *igm*, *il12*, *infγ*, *cox2*, *mhcII*).

**Figure 1:**
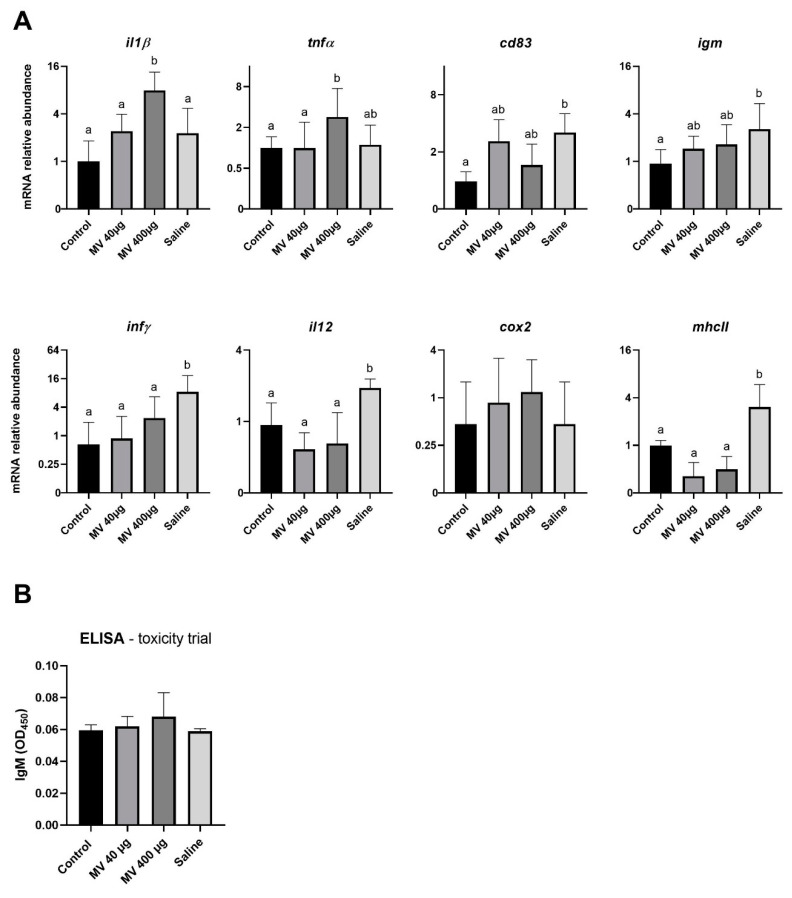
Immune responses in tilapia 14 days after injection of 40 or 400 µg of *Fo* membrane vesicles (MVs) compared to saline-injected or untreated fish. (**A**) Log2 of relative mRNA abundance of a selection of immune genes performed by qPCR on isolated spleen from eight fish per group. (**B**) Enzyme-linked immunosorbent assay (ELISA) measuring specific IgM against *Fo*. Absorbance was measured at 450 nm. Labelling with different letters (a; b) indicates significant differences between groups; groups that do not share letters are significantly different. The absence of letters indicates a lack of significant differences (*p* < 0.05; one-way ANOVA with multiple comparisons using Tukey test; error bars indicate mean ± SEM (standard error of the mean)). Note that the scale on *Y*-axis can differ in (**A**).

Two genes, *il1β* and *tnfα*, were significantly upregulated in response to injection with 400 µg of *Fo* MVs and, interestingly, the significant upregulation of *tnfα* was not significant compared to the saline-treated group (Figure 1A). Surprisingly, transcription of *infγ*, *il12* and *mhcII* were upregulated in response to the saline injection, which functioned as a placebo treatment. For *cd83* and *igm*, significant differences in expression were only detected in the control and the saline-treated fish. No significant changes in *cox2* gene expression level in response to the IP injections were detected. No significant increase in abundance of IgM specific antibodies was identified following ELISA of serum samples from fish in the toxicity trial (Figure 1B).

#### 3.1.2. MV Immunization Did Not Protect Tilapia From *Fo* Infection

**Figure 2:**
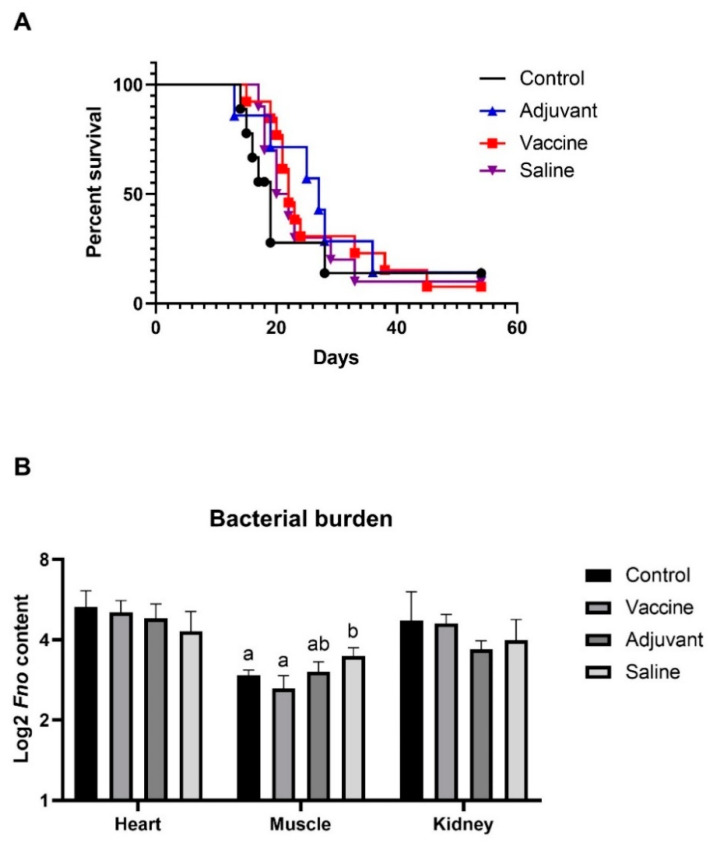
Survival and bacterial burden of adult tilapia immunized with *Fo* MVs and subsequently challenged with *Fo*. (**A**) Kaplan–Meier curve of cumulative survival (%) of tilapia immunized with 40 µg *Fo* MVs, injected with adjuvant or a saline solution, or left untreated after a cohabitation challenge with shedders injected with 3 × 10^5^ CFU/mL of *Fo*. (**B**) Graphic represents median log_2_ of *Fo* content in heart, muscle and kidney tissues collected during vaccine trial. The bacterial burden was detected by qPCR of genomic DNA isolated from the respective tissues. Different letters (a; b) indicate significant differences; groups that do not share letters are significantly different. The absence of letters indicates a lack of significant differences (*p* < 0.05; one-way ANOVA with multiple comparisons using Tukey test; error bars indicate mean ± SEM).

After establishing that *Fo* MVs did not give rise to any toxic effect, an immunization and challenge experiment was set up with the *Fo* MVs in tilapia. Although a limited immune response in the fish from the toxicity experiment was observed for the high (400 µg) dose, the dose of 40 µg *Fo* MVs was chosen for the vaccine experiment. To compensate for a reduced immune response and due to the fact that many commercial vaccine formulations include an adjuvant, the *Fo* MVs were mixed with an adjuvant before immunization. Surprisingly, no differences in survival were detected between the different groups, whether they were immunized with the MV formulation or not (Figure 2A). Aligned with these results is the observation that no significant differences in expression levels of the immune marker genes could be detected between any of the groups in the immunization trial (Figure 1A).

The bacterial burden between the various groups was then measured using gDNA, isolated from the euthanized fish (Figure 2B). In heart and kidney tissues, the detected *Fo* content was similar for all four groups. In the muscle tissue, however, a significant increase in *Fo* content was detected in the saline group compared to the control and vaccine groups, but not to the adjuvant, which was not statistically different from vaccine and control.

### 3.2. Fnn MV Toxicity and Immunization Trial in Atlantic Cod

High concentrations of Fnn MVs are not toxic and do not induce significant immune response in Atlantic cod.

**Figure A2:**
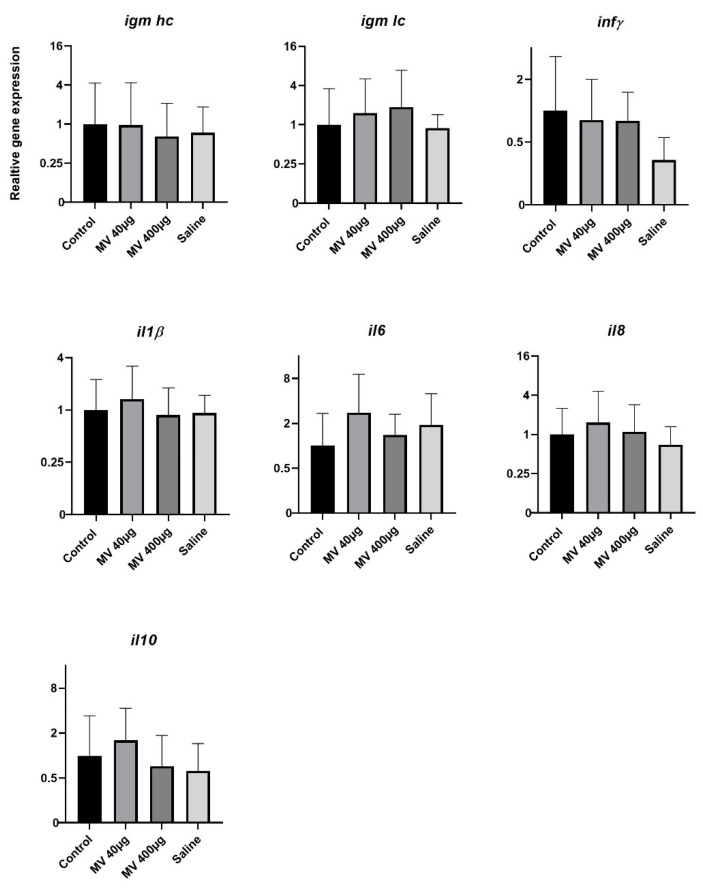
Immune genes with no significant differences in gene expression levels between the different treated groups during the toxicity trial in Atlantic cod. Gene expression level of different investigated genes were analyzed by qPCR and are displayed as relative gene expression. The gene expression levels were tested for significant differences in between the treatments (*p* < 0.05; one-way ANOVA with multiple comparison using Tukey test; error bars indicate mean ± SEM).

When injecting Atlantic cod with either the 40 or 400 µg dose of MVs, none of the fish showed any clinical signs or toxicity reaction. These results are similar to those observed in tilapia. To further evaluate the potential immune responses to the MVs in the fish, gene expression levels were monitored for a selection of immune regulating genes between the different test groups (*igm hc*, *igm lc*, *il1β*, *il6*, *il8*, *infγ*, *il10*) (Figure A2). Interestingly, none of the investigated genes showed any significant up- or downregulation in response to the different concentrations of injected MVs or the saline injection.

#### Immunization with MVs Did Not Induce Immunity against *Fnn* Infection

Similar to tilapia, Atlantic cod was immunized with a dose of 40 μg of *Fnn* MVs during the immunization and challenge trial. Also similar to tilapia, an adjuvant was used to boost the lack of immune response observed during the toxicity trial. Throughout the duration of the vaccine trial, both during the immunization and the challenge period, only three fish from different groups died. This fits the characterization of francisellosis as a chronic disease in Atlantic cod [13]. During sampling at 30 dpc, however, typical signs of infection were observed. Fish from all groups had macroscopically visible granuloma in the liver, which could in some cases also be seen on the spleen and kidney. Most of the fish had pale hearts and gills and enlarged spleens and kidneys. In some fish, a congested liver was observed in addition to the granuloma. Additionally, in some of the vaccinated and adjuvant-treated fish, fusions between liver and muscle tissues in the intraperitoneal cavity were observed.

**Figure 3:**
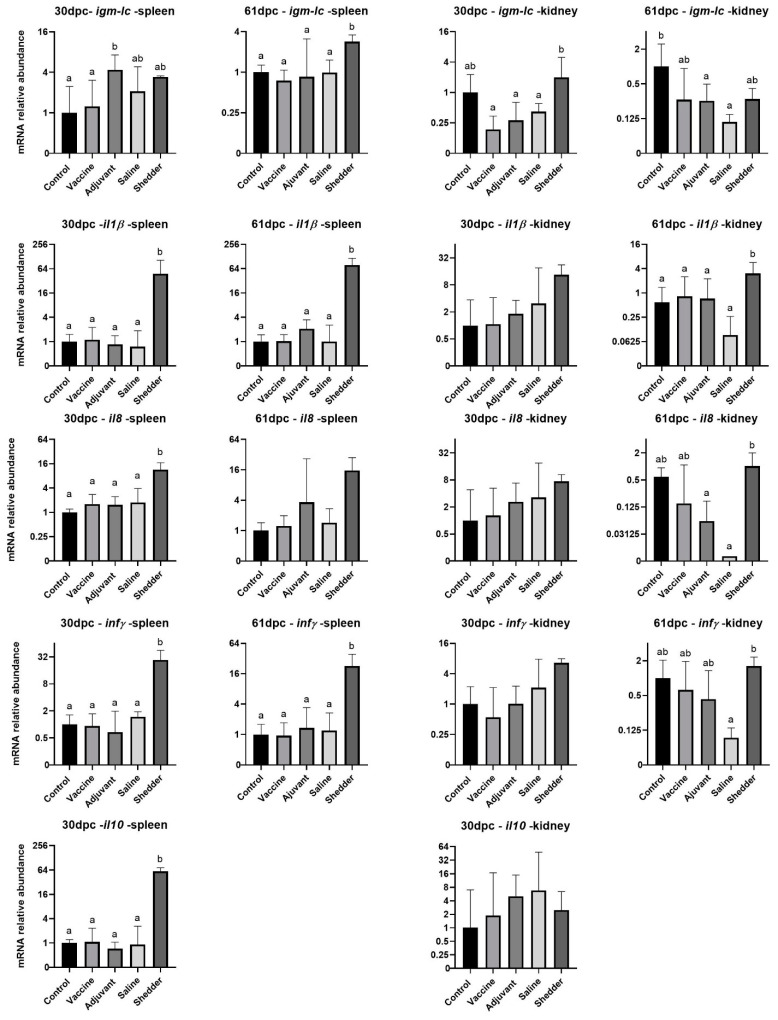
Immune gene expression levels in Atlantic cod immunized with *Fnn* MVs and challenged with *Fnn*. Sample analysis was performed by qPCR on the spleen and kidney at two time-points—30 and 61 dpc. The relative gene expression of different investigated genes displayed as log_2_ of relative mRNA abundance. Results marked with different letters (a; b) indicate significant difference; groups that do not share letters are significantly different. The absence of letters indicates the lack of significant differences (*p* < 0.05; one-way ANOVA with multiple comparisons using Tukey test; error bars indicate mean ± SEM). Note that scales on the *Y*-axis may differ.

A selection of immune genes that were found to be expressed in Atlantic cod samples (*igm*, *il1β*, *il8*, *infγ*, *il10*) was used for the analysis of immune responses by qPCR from both kidney and spleen harvested at 30 and 61 dpc. The primary immune response, detected in form of *igm* expression, showed significant upregulation in the adjuvant-treated group compared to the control and vaccine groups—30 dpc in the spleen tissue (Figure 3). Compared to the gene expression level of *igm* at 30 dpc, the shedder group showed an upregulation after 61 dpc, whereas the other groups seemed to have the same expression level. In the head kidney, *igm* expression was upregulated in the shedder group at 30 dpc and, interestingly, *igm* expression at 61 dpc was significantly downregulated in the adjuvant- and saline-treated groups compared to the control group. The expression of the proinflammatory cytokine *il1β* was constantly significantly higher in the shedder group at the different time-points in the two different tissues—a trend also observed for *il8*, *infγ* and *il10*. At 61 dpc in the kidney, *il8* was downregulated in the adjuvant- and especially in the saline-treated groups in comparison to the control and vaccinated groups. The same downregulation of gene expression was observed for *infγ* in the kidney of the saline-treated group at 61 dpc. In the case of *il10*, expression was only detected in the spleen and kidney samples that were taken at 30 dpc, following the general detected trend of higher gene expression in the shedder group, at least in the spleen tissue. In the head kidney, there were no significant differences between the different groups for the expression level of *il10*.

### 3.3. Increased IgM level in Fnn Immunized Atlantic Cod before and 30 Days Postchallenge

**Figure 4:**
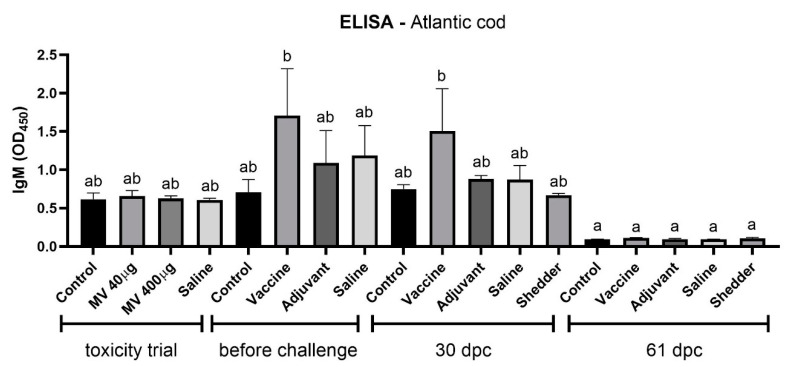
Detection of anti-*Fnn* IgM in the serum of Atlantic cod immunized with *Fnn* MVs assayed by ELISA in both the toxicity and vaccine trials. In the toxicity trial, the fish were immunized with either 40 or 400 µg of *Fnn* MVs. In the vaccine trial, the fish were immunized with either 40 µg of *Fnn* MVs mixed with an adjuvant or with adjuvant alone. In both trials, a saline-injected group was included in addition to an untreated group (control). In the toxicity trial, the serum was collected 14 days after *Fnn* MV injection. Serum samples were obtained from three fish from each respective group at given time-points during the vaccine experiment. Results marked with different letters (a; b) show the significant difference; groups that do not share letters are significantly different (*p* < 0.05; one-way ANOVA with multiple comparison using Tukey test; error bars indicate mean ± SEM).

No difference in IgM against *Fnn* could be detected in any of the groups during the MV toxicity trial in Atlantic cod (Figure 4). These samples were harvested only 14 days after immunizations and could be the reason for this lack of immune response in the fish. An increased abundance of IgM specific against *Fnn* could be detected in samples from the immunized group injected with 40 μg of MVs 50 days after immunization compared to the nonimmunized groups and the group injected only with adjuvant. The same observations were made at 30 dpc. At days 0 and at 30 dpc, the antibody levels of vaccinated fish were significantly higher than the antibody levels in all of the groups at 61 dpc.

## 4. Discussion

Francisellosis caused by *Fnn* and *Fo* has emerged to a worldwide problem in fish farms and efforts have been made to find an effective vaccine. The vaccine candidates tested in this study are isolated MVs from *Fnn* and *Fo*, as MV vaccines against francisellosis showed promising results when previously tested in an adult zebrafish model [26,27]. It was therefore of interest to investigate the possible vaccine application of isolated MVs in the natural hosts of *Fnn* and *Fo*, the Atlantic cod and tilapia, respectively. Interestingly, but unfortunately, a protective immunity could not be observed in the natural hosts, the Atlantic cod and tilapia, when the fish were immunized with MVs isolated from *Fnn* or *Fo*. When initially testing the potential toxicity of the MVs in the fish, an interesting result in tilapia was the upregulation of the proinflammatory cytokine *il1β* in the spleen of fish injected with 400 µg of MVs. This upregulation resembles the immune response seen in the actual bacterial infection [40,41]. This could indicate that the acellular MVs can initiate an immune reaction in tilapia, as previously shown in zebrafish [27]. In comparison to this, *infγ* was only significantly upregulated in saline-treated tissue, whereas in zebrafish injected with *Fo* MVs, it was significantly upregulated [27]. This could be an indication of the different effects of MVs on gene expression in its natural host and the model fish. It was previously shown that *Francisella ssp.* have immune-suppressive influences and interfere with *infγ* signaling. It is proposed that *Francisella ssp.* target the *infγ* signaling pathway by preventing of downstream activation of transcription factors [42,43]. LC-MS/MS analysis of the MV content of *Fo* showed an abundance of important virulence factors, such as IglC (a protein necessary for intracellular growth and escape from phagolysosomes), chaperon proteins associated with immunogenic effects and cytoplasmic proteins. This cargo could be sufficient to influence the host immune response [27]. Another observation was that *mhcII*, *infγ* and *il12* are significantly upregulated in the saline-treated group compared to the control group. In a different study that used an inactivated whole-cell vaccine, an upregulation of *mhcII* expression level was observed after vaccination, which was associated with the successful recognition of the *Fo* cells [44]. In the case of the injected *Fo* MVs, the upregulation of the proinflammatory cytokines but not *mhcII*, *infγ* or *il12* expression, which are only upregulated in the saline-treated group, supports the assumption that MVs are capable of active manipulation of the immune response in a similar fashion to natural infections. Further supporting this theory, *igm* gene expression is only upregulated in the saline-treated group compared to the control group. Upregulation of *igm* gene expression and the detection of *Fo* specific IgM in serum samples was reported in a previous study and is associated with B-cell activation which is involved in development of a protective immune response [44]. In zebrafish, *igm* expression was also significantly upregulated 7 days post vaccination with *Fo* MVs [27]. The immune response of zebrafish, used as a model organism, and tilapia, the natural host, might of course differ. In accordance with the lack of induction of *igm* mRNA in the spleen, no significant amount of IgM antibody against *Fo* was found in the tilapia serum. The expression level of the B-cell marker *cd83* is, as *igm*, only upregulated in the saline-treated group compared to the control group. When looking at differences in response to the two different doses of *Fo* MV, only the significant upregulation of the genes coding for the proinflammatory cytokine *il1β* was detected. Generally, there do not seem to be any further detectable differences in gene expression levels in response to the different dosages of immunization with the MVs. These data indicate that the injected *Fo* MVs do not have any toxic effect on tilapia and that the cargo of the MVs might have the ability to actively influence the immune response in this fish species.

The general upregulation of genes in the saline-treated group leads to the assumption that the IP injection itself also triggers an immune response. It is a forced invasion into the fish body and the penetration through skin and tissue into the intraperitoneal cave could enable other pathogens to enter. However, the IP injections were performed under aseptic conditions so that the increased expression levels occur most likely as an immune response to the stress induced by anaesthesia, handling and injection of saline. Usually, the saline-treated group serves as the control group, but we included a control group without any treatment in our experiments. This control group enabled detection of the upregulations in the saline-treated group, indicating a possible effect on the immune response being triggered by the IP injection alone.

Previous trials with MVs of Gram-negative bacteria as vaccine candidates showed promising results—i.e., against edwardsiellosis in olive flounder (*Paralichthys olivaceus*) or against infection with *Flavobacterium psychrophilum* in rainbow trout (*Oncorhynchus mykiss*) [31,45]. In this study, tilapia were vaccinated with 40 µg of *Fo* MVs and the fish were kept for 24 days, in order to develop specific immunity, before they were challenged by *Fo* infection. The survival rate displayed no significant difference between the different groups and there was no vaccine efficacy detectable. Within vaccinated groups, qPCR analysis did not reveal any significant up- or downregulation of any of the tested immune genes. This might be due to the sampling time-point, which was after the trial at 61 dpc, where possible changes in expression levels were most likely not detectable. For future trials, several and earlier sampling time-points are eligible, such as, i.e., sample points for zebrafish, or in tilapia [27,44]. Significant differences in bacterial load were only detected in the muscle tissues of the different groups. The control and the vaccinated groups had a significantly lower bacterial load than the saline-treated group, which may be an indication for an impaired innate immune system in the vaccinated group, caused by the reaction in response to the vaccine injection, and that no immunity was formed to the sampling time-point. Similarly, it was expected that the bacterial burden would be similar in the control group and the group of fish injected with saline solution. This result aligns with the observation that the MVs might not induce the development of protective immunity against *Fo* infection in tilapia. The MVs seem to interfere and manipulate the host immune system similar to the pattern of the bacteria itself, by upregulation of certain proinflammatory cytokines and prevention of downstream transcription factor activation [34,42,43,44,46,47,48,49]. Besides this, an adjuvant was used in combination with the MVs. Thus, it cannot be ruled out that the adjuvant did not have the expected effect of enhancing an immune response and development of specific immunity together with the MVs, but rather the opposite. The group treated only with adjuvant did not display an elevated immune response nor improved immunity against the infection compared with the MV-treated group, suggesting no beneficial effect of using an adjuvant.

In contrast to the toxicity trial in tilapia, the results from the toxicity trial in Atlantic cod with *Fnn* MVs showed no significant up- or downregulation in any of the tested genes. It is possible that the sampling point 14 days after injection would not reveal any specific immune response, neither early nor late, respectively. The sampling could have been performed earlier or the experiment should have been continued longer. In any case, several sampling points would be preferable in order to detect comprehensive changes in the gene expression levels.

Francisellosis in Atlantic cod is characterized as a chronical, nonlethal infection [13] and during the time-span of the vaccine trial, no disease-related mortalities were detected. However, observations during sampling of the fish at 30 dpc described typical signs of francisellosis infection. The described granuloma on liver, spleen and kidney tissues in addition to occasionally observed enlargement of spleen and kidney, in fish from all groups, indicate that the MVs used as a vaccine were not able to prevent *Fnn* from infecting the vaccinated fish. Additionally, in some of the vaccinated and adjuvant-treated fish, fusions between liver and muscle tissues in the intraperitoneal cavity were observed. The fusions are likely a result of the IP injection which was either unfortunately placed or the adjuvant might have negatively interfered with the tissue and induced the formation of lesions [50].

Samples collected from shedder fish were included in the analysis for this trial and provided an interesting comparison. When analyzing the immune gene expression of both spleen and kidney, only *il1β* was significantly upregulated in the shedder group compared to the control, except for 30 dpc in the kidney. This gene regulation of *il1β* expression levels resembled the known immune response pattern to *Fnn* infection [40,51]. The fact that it is not upregulated in any of the other groups is, however, surprising since one would expect the other fish in the tank to be infected with francisellosis at the sampling time-points. *infγ* expression levels are upregulated in the shedder’s spleen; however, from viewing previously described expression levels of *infγ* during infection in Atlantic cod, one would rather expect it to be downregulated [42,43,51]. The expression level of *igm* varies in-between sampling time-points and tissue. The upregulation of *igm* in the adjuvant group at 30 dpc in the spleen, compared to the vaccinated and control group, displays the desired effect of the adjuvant—i.e., to enhance an immune defense. At 30 dpc in the kidney, *igm* was upregulated in the shedder group compared to the vaccine, adjuvant and saline groups. This might be due to the long exposure and infection times in the shedders, which were directly injected with the pathogen. At 61 dpc in the spleen sample, an increased *igm* level was detected in the shedders, compared to all other groups. This might be due to the direct infection dose injected into the shedders. In comparison to this, *igm* is significantly upregulated in the control group in the kidney at 61 dpc and seems therefore downregulated in the adjuvant- and saline-treated groups. More sampling time-points are important in order to trace changes in gene expression comprehensively. To the two time-points of the vaccine trial, another sampling before the challenge would be preferable. IgM levels in serum samples were only significantly upregulated in the vaccinated group before the challenge and at 30 dpc in comparison to all samples at 61 dpc. Interpreting these results as an indication for induced immune response in response to the vaccine seems bold as there is no significant difference to the other samples. However, the early increase in specific IgM levels resembles results from a current MV trial in salmon (*Salmo salar*) where an early short-term immunization could be observed (data not published).

Taken together, there are few indications that vaccination at a dose of 40 µg of *Fnn* or *Fo* MVs induces protection against francisellosis. The immunized fish were not found to reveal any significantly improved immune response towards the challenge with these two infectious agents. The macroscopic observations made during sampling, however, led to the belief that there would not have been a detectable difference. A higher dose of MVs could induce an immune response that would lead to protective immunity. The doses applied in this study were, however, the same as previously used in zebrafish [26]. Other studies using isolated pathogen MVs in fish and mammals showed induction of immunity with even lower injection doses (5–20 µg of MV concentration) [27,31,38,39]. This indicates that the applied MV doses should be evaluated for the respective pathogen they are being isolated from. However, viewing the results of the MV injection in tilapia, the effect of a higher MV dose might only lead to suppression and manipulation of the immune system rather than inducing the development of immunity. The immune system of Atlantic cod in itself is quite unique, considering the lack of genes coding for mhcii and needed components for its expression and transportation [52]. The diversity in the immune system between Atlantic cod and tilapia might account for differential gene expression in response to MV injection.

It would be an interesting experimental setup to test many different concentrations of MVs in both Atlantic cod and tilapia and further evaluate and compare the impact on the immune response. A different immunization approach could involve mixing of the MVs with inactivated bacteria, as has been performed for *Flavobacterium psychrophilum* in rainbow trout (*Oncorhynchus mykiss*) [45]. As mentioned before, more sampling time-points and a longer immunization phase should be considered. Immune studies in carp (*Cyprinus carpio*) suggested that formation of optimal immunological memory is reached only after three to eight months and Ye et al. (2013) suggested that, for the development of successful vaccines in fish, more studies on development of long-term memory are required [53,54].

## 5. Conclusions

The interesting finding in this study is that no protective immunity was accumulated against francisellosis in two species—Atlantic cod and tilapia—when immunized with MVs isolated from *Fnn* and *Fo*, respectively. This lack of induced immunity is in stark contrast to previously performed MV immunization experiments in olive flounder (*Paralichthys olivaceus*), rainbow trout (*Oncorhynchus mykiss*) or zebrafish (*Danio rerio*) [26,31,45]. Taken together, it is thus not possible to conclude that MVs would be reasonable vaccine candidates against francisellosis in fish.

It would be interesting to explore further the induced and possibly suppressed pathways and gene regulations involved in the immune response to MVs in these different hosts. It would also be especially interesting to understand the detailed differences between the responses towards the respective pathogens in the respective warm and cold water hosts, meaning that further in vivo studies should be conducted to unravel the factors that determine the development of immunological memory in fish.

## Figures and Tables

**Figure A1:**
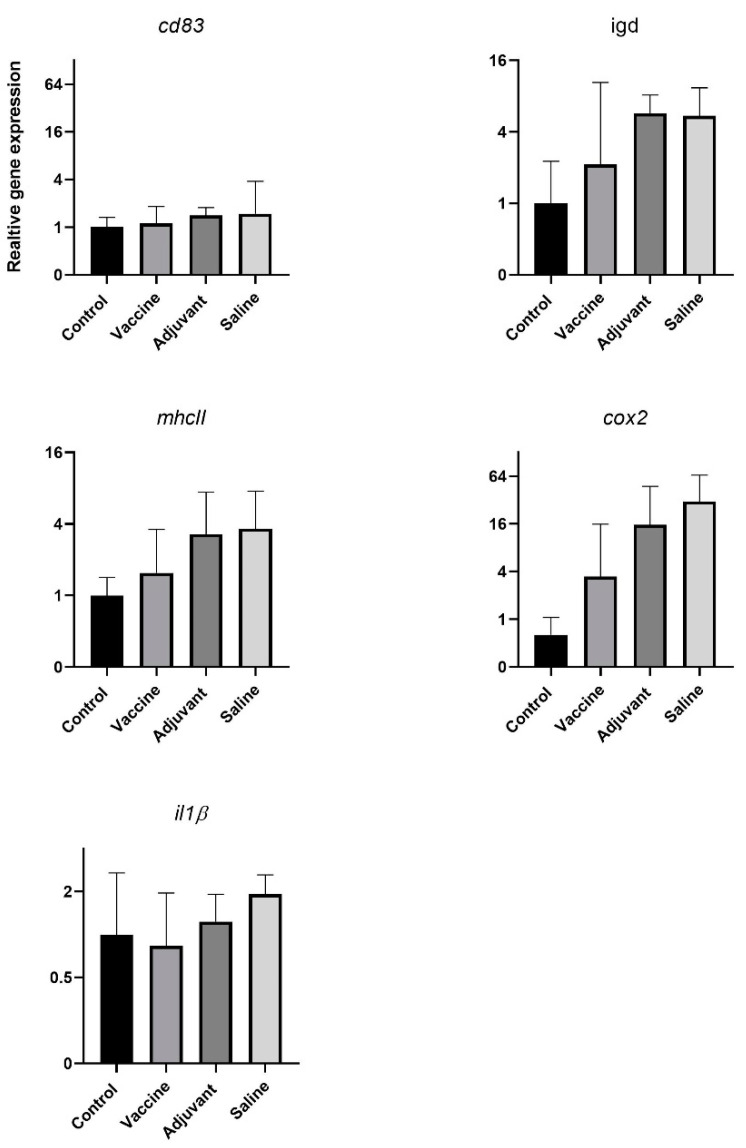
Immune genes with no significant differences in gene expression levels between the different treated groups during the vaccine trial in tilapia. Gene expression levels of the investigated genes were analyzed by qPCR and are displayed as log_2_ of relative gene expression. The gene expression levels were tested for significant differences in between the treatments; (*p* < 0.05; one-way ANOVA with multiple comparison using Tukey test; error bars indicate mean ± SEM).

## Data Availability

The data presented in this study are available in this article and in the Appendix A material.

## Appendix A

**Table A1:** List of primer used for qPCR and bacterial burden.

Name	Sequence 5′−3′	Reference
CD83_Tilapia_fw	CCGGTGTCCAGTACCTTGCAGTGA	[55]
CD83_Tilapia_rv	GCTGGACAATCTGCCAGCACCAG	
IgD_Tilapia_fw	AACACCACCCTGTCCCTGAAT	[56]
IgD_Tilapia_rv	GGGTGAAAACCACATTCCAGC	
Tnfα_Tilapia_fw	TCATGGCTGGTATAACGCGA	This study
Tnfα_Tilapia_rv	GTACGGCCTTCATCGGTTTC	
Il-12_Tilapia_fw	GGAAGCACGGCAGCAGAATA	[57]
Il-12_Tilapia_rv	AACTTGAGGGAGAAGTAGGAATGG	
IFN- γ_Tilapia_fw	AGCGGCTGACTGAACTCAATTGAAG	[57]
IFN-γ_Tilapia_rv	GTCACAGTTTTCAGCTGTATAGGG	
β-actin_Tilapia_fw	CAGGATGCAGAAGGAGATCACA	[58]
β-actin_Tilapia_rv	CGATCCAGACGGAGTATTTACG	
IgM_Tilapia_fw	AGGCACAACGGTCACTGTCA	[59]
IgM_Tilapia_rv	GCAAGGCAGCCAAGAGTGAC	
MHCIIb_Tilapia_fw	GAGGAACAAGCTCGCCATCG	[59]
MHCIIb_Tilapia_rv	AGTCGTGCTCTGACCTCGAG	
COX2_Tilapia_fw	AGCAGCCAGAAGGAAGGCGG	[60]
COX2_Tilapia_rv	GACTGAGTTGCAGTTCTCTTAGTGTGC	
Il-1β_Tilapia_fw	TGCTGAGCACAGAATTCCAG	[61]
IL-1β_Tilapia_rv	GCTGTGGAGAAGAACCAAGC	
Ef1α_Cod_fw	ATGTGAGCGGTGTGGCAATC	[62]
Ef1α_Cod_rv	TCATCATCCTGAACCACCCTG	
Ubi_Cod_fw	GGCCGCAAAGATGCAGAT	[63]
Ubi_Cod_rv	CTGGGCTCGACCTCAAGAGT	
IL1β_Cod_fw	GGAGAACACGGACGACCTGA	[62]
IL1β_Cod_rv	CGCACCATGTCACTGTCCTT	
IL6_Cod_fw	TGAAGAAGGAGTACCCCGACAAT	[48]
IL6_Cod_rv	GGTGCCTCATCTTTTCCTCAATG	
IL8_Cod_fw	GGTTTGTTCAATGATGGGCTGTT	[62]
IL8_Cod_rv	GACCTTGCCTCCTCATGGTAATACT	
IL10_Cod_fw	CCTATAAAGCCATCGGCGAGTTA	[62]
IL10_Cod_rv	TGAAGTCGTCGTTTTGAACCAAG	
INF-γ_Cod_fw	TGGTCTGCATGTCAGTTTGTCTG	[62]
INF-γ_Cod_rv	TTCTGTGGATGTTGTTGGCTAAGA	
IGM-LC_Cod_fw	CACTACAGCTGGAGCAGCAC	[64]
IGM-LC_Cod_rv	CCATGCTGGAGCCTCTCTAC	
IGM-HC_Cod_fw	GGTGAGGTGTTATCCGTGCT	[64]
IGM-HC_Cod_rv	GCAGATAAACGGATGGAGGA	
Fo fw	CATGGGAAACAAATTCAAAAGGA	[37]
Fo rv	GGAGAGATTTCTTTTTTAGAGGAGCT	
